# Optimizing Salt Concentration for Reliable Aqueous Size-Exclusion Chromatography of Water-Soluble Polymers

**DOI:** 10.3390/polym18131571

**Published:** 2026-06-24

**Authors:** Lilian Lin, Gregory T. Russell, Heon E. Park

**Affiliations:** 1 Department of Chemical and Paper Engineering, Western Michigan University, Kalamazoo, MI 49008, USA; lilian.lin@wmich.edu; 2 School of Physical and Chemical Sciences, University of Canterbury, Christchurch 8041, New Zealand; greg.russell@canterbury.ac.nz

**Keywords:** gel-permeation chromatography (GPC), sodium alginate, dn/dc, water-soluble polymer, anionic polymer, light scattering (LS), polymer–column interactions, molecular weight, non-size-exclusion interactions, optimized salt concentration

## Abstract

Size-exclusion chromatography (SEC) or gel-permeation chromatography (GPC) is an essential tool for determining the molecular weight and polydispersity of water-soluble polymers, including biopolymers used in hydrogels, sealants, bioinks, and other biomedical materials. However, aqueous SEC of polyelectrolytes, i.e., charged polymers, is often complicated by non-size interactions among polymer chains, porous column beads, pore surfaces, frits, tubing, and mobile phase. Salt addition to eluent is commonly used to screen these interactions, but the minimum salt concentration required to restore reliable SEC behavior remains poorly defined, and excessive salt may introduce tailing, refractive-index artifacts, deposits, or instrument concerns. In this study, aqueous SEC with refractive index (RI) and right-angle light scattering (RALS) detection was used to evaluate the effect of salt ($Na_{2}SO_{4}$) concentration on poly(ethylene oxide) (PEO), a nominally neutral reference standard polymer, and sodium alginate as a model anionic biopolymer. PEO retained a single bell-shaped peak across the tested salt range, but its elution volume and SEC/RALS-derived molecular weights varied slightly with salt concentration, showing that even a nominally neutral reference polymer is affected by mobile-phase conditions. Alginate showed much stronger salt dependence: eluent at very low salt concentration produced broad, noisy, and convoluted chromatograms, whereas increasing salt concentration progressively narrowed the main peak. The first condition that produced a clear, approximately symmetric RI/RALS main peak was $6.25\times 10^{-3}\;M$ $Na_{2}SO_{4}$, identifying it as the minimum effective salt concentration for this alginate/column/instrument system. To rigorously validate these observations, we propose a set of both qualitative and quantitative peak analyses that objectively confirm the optimal mobile-phase conditions. Ultimately, these results provide a practical workflow for identifying the minimum effective salt concentration required for reliable SEC analysis of water-soluble polymers.

## 1. Introduction

### 1.1. The Need for Reliable Molecular-Weight Characterization

Water-soluble biopolymers and their chemically modified derivatives are increasingly used as advanced materials for tissue adhesives, sealants, injectable hydrogels, drug-delivery matrices, wound dressings, cell-laden scaffolds, and bioinks. Examples include alginate- and gelatin-based pleural and tracheal sealants [1,2], gelatin methacryloyl (GelMA) hydrogels whose gelation and photocrosslinking depend on synthesis and processing conditions [2,3], methacrylated hyaluronic acid (HAMA) and hybrid GelMA/HAMA scaffolds for tissue engineering [4,5], dextran-methacrylate hydrogels for biomedical applications [6], pectin-based hydrogels for biomedical uses [7], oxidized and epimerized alginates for biofabrication and structure-property studies [8,9], peptide-modified alginate bioinks [10], chitosan methacrylate and catechol-modified chitosan hydrogels for antibacterial or adhesive wound dressings [11,12], methacrylated gellan gum inks for extrusion bioprinting [13], methylcellulose/GelMA bioinks [14], and methacrylated cellulose nanofiber hydrogels [15]. Molecular weight is a critical characteristic of these polymers, as it profoundly influences the macroscopic properties of the final products. Consequently, it is expected that the molecular weight will be altered during the modification process. Hence, precise molecular-weight characterization is a fundamental prerequisite for the successful design, engineering, and manufacturing of biopolymeric products.

### 1.2. The Role of Molecular Weight in Material Performance

Molecular weight is one of the most consequential descriptors in this field because it influences chain entanglement, solution viscosity, rheology, printability, gelation kinetics, crosslink density, degradation rate, diffusional transport, tissue retention, and mechanical performance. During chemical modification, purification, sterilization, or storage, molecular weight can change unintentionally because of hydrolysis, fractionation, oxidation, radical-induced scission, incomplete recovery of high-molecular-weight fractions, or aggregation. Reliable molecular-weight characterization is thus essential not only to confirm successful synthesis, but also to interpret why one batch forms a stronger hydrogel, a more adhesive sealant, a more stable bioink, or a more reproducible biomedical polymer network than another [1,3,4].

### 1.3. Size-Exclusion Chromatography as a Characterization Tool

Size-exclusion chromatography (SEC), also commonly called gel-permeation chromatography (GPC) in polymer science, is one of the most important techniques for this purpose. In ideal SEC, macromolecules are separated according to hydrodynamic volume rather than chemical affinity. A column is packed with a stationary phase of porous beads, and larger molecules are excluded from a greater fraction of these pores, consequently exhibiting earlier elution, whereas smaller molecules permeate more of the pore volume and consequently elute later. By employing a multi-detector configuration that combines concentration-sensitive detectors such as refractive index (RI) or ultraviolet (UV) absorption with molecular-weight-sensitive light-scattering detectors, such as right-angle light scattering (RALS) or low-angle light scattering (LALS), the method can provide relative elution profiles and slice-by-slice molar-mass information if detector calibration and specific refractive index increment (dn/dc) handling are appropriate [16,17]. This is attractive for biomedical polymers because one chromatogram can reveal average molecular weights, polydispersity, low-molecular-weight fractions, multimodality, degradation, traces of impurities, or aggregation.

In this manuscript, the term SEC is used preferentially because IUPAC defines SEC as separation mainly according to hydrodynamic volume in a porous non-adsorbing material, whereas GPC is defined as a form of SEC in which the porous non-adsorbing material is a gel [18]. The term GPC is retained where appropriate because it remains widely used in polymer characterization.

### 1.4. Challenges in the Aqueous SEC of Charged Biopolymers

The main difficulty is that aqueous SEC is seldom ideal for charged biopolymers because many behave as polyelectrolytes in aqueous mobile phases (water). Polyelectrolytes such as alginate, hyaluronic acid, heparin, pectin, carrageenan, poly(acrylic acid), proteins, and chitosan derivatives can experience intramolecular repulsion, electrostatic exclusion from pores, weak adsorption at pore entrances, hydrogen bonding, polar interactions with methacrylate-based stationary phases, and interactions with metallic frits or tubing. As a result, the observed chromatogram can be broader, narrower, shifted, tailed, split, or otherwise distorted relative to a purely size-exclusion separation [19,20]. Interaction chromatography intentionally exploits such non-size forces [21], but in conventional SEC, they are artifacts that must be minimized. More detailed mechanistic descriptions are provided in the next section.

### 1.5. Polyelectrolyte–Column Interactions and Role of Salt in Aqueous SEC

**Figure 1:**
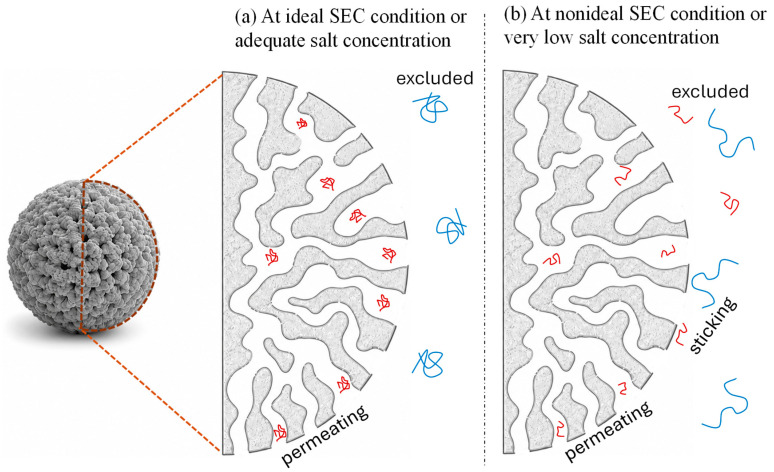
Salt-dependent transport of anionic polymer chains through or next to a porous bead in aqueous SEC. The spherical bead image on the left was generated using ChatGPT 2.52 as a generic visual representation of a porous chromatographic bead. All other schematic elements were prepared and edited by the authors. The figure is schematic, not drawn to scale, and should not be interpreted as an experimental micrograph or as a quantitative representation of the actual pore structure. (**a**) Under ideal SEC conditions or with adequate added salt, electrostatic interactions are screened and chains adopt a smaller screened-coil conformation, allowing more size-controlled pore access. Short chains in red color permeate deeply, and long chains in blue color remain mostly excluded. (**b**) Under nonideal SEC conditions or with insufficient salt, low ionic strength causes electrostatic chain expansion and heterogeneous polymer–bead interactions. Some chains are excluded from pores, whereas others adsorb at bead surfaces or pore mouths, broadening the elution-time distribution.

In ideal aqueous SEC, macromolecules are separated primarily by hydrodynamic volume. Larger chain coils access less pore volume and elute earlier, whereas smaller coils penetrate more pores and elute later (Figure 1a).

For polyelectrolytes, however, this size-based mechanism can be disrupted because the charges that make the polymer water-soluble also affect chain conformation and interactions with the stationary phase. In low-ionic-strength eluents such as DI water, intramolecular repulsion among charged repeat units expands the polymer coil, increases the effective hydrodynamic volume, and can reduce pore access (Figure 1b). Charged-polymer partitioning in SEC is therefore governed by both steric size and electrostatic free energy, not by molecular size alone [19]. Polyelectrolyte theory similarly describes chain stiffness as having an electrostatic contribution that decreases with increasing ionic strength [19]. The stationary phase further complicates aqueous SEC of polyelectrolytes. Many aqueous SEC columns use hydrophilic polymeric packings, including cross-linked polymethacrylate, PMMA-type, hydroxylated polymethacrylate, or related bead chemistries. Although PMMA and polymethacrylate packings are often treated as nominally neutral compared with ion-exchange resins, real aqueous bead surfaces are not perfectly inert. PMMA particles and surfaces can show measurable electrokinetic charge in water, and this charge can depend on electrolyte, pH, surface history, and adsorbed species [19,22]. In addition, polymeric beads can contain residual ionic end groups or charged/polar surface sites from initiators, stabilizers, hydrolysis, or manufacturing chemistry; PMMA particles with ionic end groups have been shown to develop measurable mobility in water [23], and hydrolysis of methacrylate-type particles can generate surface carboxyl groups [24], which are one of the most common functional groups in biopolymers.

As a result, polyelectrolyte chains can experience several non-SEC interactions with bead surfaces, pore walls, frits, and other wetted components (Figure 1b). If polymer and surface charges repel each other, the polymer may be excluded from pore entrances and elute earlier than expected. If local attractive, polar, hydrogen-bonding, hydrophobic, or ion-mediated interactions occur, part of the polymer population may adsorb transiently and elute later, producing tailing or shoulders. Aqueous SEC studies of polyelectrolytes show that mobile-phase and stationary-phase chemistry strongly influence such non-size interactions [20], and SEC reviews identify ionic adsorption, secondary interactions, retention shifts, broadening, and peak asymmetry as common causes of nonideal chromatograms [16]. This low-salt behavior affects different chain lengths differently. Long chains may already be largely excluded from many pores because of their intrinsic hydrodynamic size, so electrostatic expansion mainly reinforces early elution. Short and medium chains normally rely on pore entry for SEC separation. At the same time, a fraction of chains can adsorb or desorb slowly from bead surfaces or pore mouths and elute later. The coexistence of electrostatic expansion, pore exclusion, and adsorption/desorption broadens the elution-time distribution and can generate shoulders, tailing, or convoluted peaks [19,20].

Adding salt to the eluent increases ionic strength and screens electrostatic interactions. Counterions reduce the range of charge–charge repulsion along the polymer chain (i.e., intramolecular repulsion), decreasing electrostatic expansion and allowing the chain to approach a more compact screened-coil conformation. Salt also screens interactions between charged polymer segments and charged or polar sites on the stationary phase, reducing heterogeneous pore exclusion and adsorption/desorption residence-time effects. Under an appropriate salt condition, short chains can enter pores more reproducibly, medium chains show more uniform partial pore access, and long chains remain largely excluded according to hydrodynamic size. The resulting separation is therefore closer to ideal SEC (Figure 1a), with a narrower and more symmetric main peak [19,20].

However, the highest salt concentration is not necessarily optimal. Once electrostatic expansion and electrostatic polymer–surface interactions are sufficiently screened, additional salt may provide little further improvement in size-based separation. Instead, high salt can introduce or enhance other non-SEC effects, including residual hydrogen bonding, polar or hydrophobic interactions with bead surfaces, salting-out tendencies, altered polymer hydration, metal-surface interactions, RI mismatch, baseline disturbances, or salt-related injection artifacts. Hence, a high-salt eluent may still produce right-side tailing or late-eluting features even when the dominant electrostatic polyelectrolyte effect has been suppressed [16,20]. For this reason, aqueous SEC of water-soluble polyelectrolytes should be optimized using a minimum-effective-salt strategy: the selected eluent should contain enough salt to restore reproducible, size-controlled transport, but not so much salt that secondary interactions, detector artifacts, or operational problems become significant.

### 1.6. Current Literature and Study Objectives

It is crucial to elucidate the role of salt additives and employ the minimum effective concentration necessary to achieve reliable chromatographic separations. Table 1 summarizes the common salts/additives used in aqueous SEC, detailing their typical functions, practical advantages, limitations, and hardware implications. Previous quantitative SEC studies for water-soluble polymers have established the fundamental role of ionic strength and column chemistry. For example, Ci et al. developed high-performance SEC for sodium alginate molecular-mass distributions [25]. Vold et al. used SEC with light scattering and viscosity detectors to study alginates, epimerized alginates, and periodate-oxidized alginates [8]. Díaz Baños et al. directly examined the influence of ionic strength on alginate flexibility using SEC [26]. Caltabiano et al. studied aqueous SEC of the synthetic polyelectrolyte sodium polystyrene sulfonate on reversed-phase (RP) chromatography and hydrophilic interaction liquid chromatography (HILIC) columns [20]. More recently, Vidal et al. varied aqueous SEC conditions, including ionic strength, pH, temperature, and column arrangement, for polyethylene glycol (PEG), PEO, and sodium polyacrylate calibration curves [27]. These studies confirm that ionic strength and column chemistry matter. ASTM F2605-16(2025) [28] specifies a single salt concentration of 0.05 M for the size-exclusion chromatography (SEC) analysis of sodium alginate. Recent SEC tutorials and alginate-specific fractionation studies further emphasize that molecular-weight values from separation/light-scattering methods depend on detector assumptions, aggregation state, dn/dc handling, and data-processing choices [17].

However, practical guidelines for optimizing salt concentrations remain scarce, particularly for polyelectrolytic systems. This challenge is compounded by the fact that even nominally neutral reference standards, such as poly(ethylene oxide) (PEO), frequently exhibit nonideal enthalpic interactions in aqueous media, thereby skewing molecular-weight calibrations. To resolve these interconnected challenges, the present study implements a comprehensive evaluation strategy.

The objectives of this work were fourfold: (i) to determine how salt concentration alters the chromatographic profiles of nominally neutral reference standards, such as PEO, (ii) to evaluate the effect of ionic strength on polyelectrolyte elution behavior across finely resolved concentration increments, (iii) to apply both qualitative and quantitative assessments to yield a well-resolved SEC peak (or envelope) while avoiding high-salt artifacts, and (iv) to establish a practical workflow for identifying the minimum effective salt concentration, generalizing this approach for application to other biopolymers and alternative aqueous SEC column chemistries.

## 2. Materials and Methods

### 2.1. Materials

Poly(ethylene oxide) (PSS-PEO46k) with weight-average molecular weight ($M_{w}$) of 46 kg/mol was sourced from Agilent (Waldbronn, Germany) and used as the standard reference polymer. Sodium sulfate (${Na}_{2}{SO}_{4}$, anhydrous, ≥99% ACS reagent) was sourced from Sigma-Aldrich (St. Louis, MO, USA). Alginic acid sodium salt (alginate, medium viscosity, product A2033, CAS 9005-38-3) was obtained from Sigma-Aldrich (St. Louis, MO, USA). The alginate, derived from brown algae, was characterized by a viscosity ≥ 2000 mPa s (2% aqueous solution at 25 °C) and a composition of approximately 61% mannuronic acid and 39% guluronic acid (M/G ratio of 1.56) [32]. While a non-official report suggests a molecular weight of approximately 200 kg/mol [33], literature values obtained via RI-only detection have been reported as high as $M_{w}=507\;kg/mol$ [34]. Deionized (DI) water was used to prepare eluents and solvents containing ${Na}_{2}{SO}_{4}$ at concentrations ranging from 0 to 0.1 M.

### 2.2. SEC Instrumentation, Detectors and Columns

A TDA305 triple-detector module coupled to a GPCmax pump/autosampler (Malvern, PA, USA) was used. More detailed operational conditions are shown in Appendix A. Although the detector train contains RI, RALS, LALS, and differential viscometry capabilities, the present quantitative analysis focused on RI and RALS. Two aqueous columns, designated A4000 and A3000, were connected in series. These columns represent two different pore regimes, with the A3000 covering lower-to-intermediate hydrodynamic sizes and the A4000 extending the usable range toward larger water-soluble polymers. The paired-column arrangement broadens the working separation range so that both PEO and alginate can be observed within the same method, although exact coverage depends on polymer chemistry and hydrodynamic volume, not molecular weight alone. The instrumental configuration and chromatographic parameters for the SEC system are detailed in Table A1. The exclusion molecular-weight limits of the columns are provided by the manufacturer based on pullulan exclusion limits and are used here only to describe column selection. Actual separation of PEO and alginate depends on hydrodynamic volume, polymer chemistry, salt concentration, and secondary interactions.

RALS was prioritized over LALS because the samples of interest were in a moderate-molecular-weight regime, while low-angle signals are more sensitive to dust, rare aggregates, stray light, alignment errors, and baseline disturbances. This was important for alginate at low salt, where scattering traces were already noisy. For larger radius of gyration ($R_{g}$) polymers or highly accurate angular extrapolation, full multi-angle treatment including LALS would be more important [17].

### 2.3. Eluent and Sample Preparation

The aqueous mobile phase was prepared at 14 distinct sodium sulfate concentrations in deionized (DI) water, as detailed in Table A2 and Table A3. The ionic strength for ${Na}_{2}{SO}_{4}$ solution, can be obtained by

(1) $$I=\frac{1}{2}\sum c_{i}z_{i}^{2}$$

where I(M) is the ionic strength of the solution, $c_{i}(M)$ is the concentration of ion i, and $z_{i}$ is the number of charges of ion i. For $Na_{2}SO_{4}$, $I=\frac{1}{2}[2c\times (1^{2})+c\times (2^{2})]=3c$, where c(M) is the concentration of the salt in the solution. Particulates were removed via vacuum filtration through a 0.22 μm cellulose acetate membrane (Corning, NY, USA). Dissolved gases and air bubbles were eliminated via ultrasonication (DT 1028 F, Bandelin electronic GmbH & Co. KG, Berlin, Germany) for 20 min.

Sample solutions were prepared by dissolving the polymer in 10 mL of the corresponding eluent in a 20 mL vial at 20 °C. To ensure complete dissolution and minimize solvent mismatch effects, each alginate sample was dissolved overnight under magnetic stirring at room temperature in the corresponding eluent, which was chemically identical to the mobile phase used for its analysis to minimize solvent mismatch, i.e., system peaks [35,36].

All polymer solutions [0.5% (*w*/*v*) PEO46k and 0.1% (*w*/*v*) alginate] were filtered through a 0.22 μm polyethersulfone (PES) membrane syringe filter (Millex^™^, Sigma Aldrich, St. Louis, MO, USA) before being transferred to the SEC sample vials. Because the filter can retain both liquid and polymer, the true injected concentration was not assumed to be identical to the nominal preparation concentration. To estimate the true concentration, 2 mL of the filtered sample was reserved for SEC, and the remainder was weighed in the original capped vial using a microbalance (Quintix 65-1S, Sartorius, Göttingen, Germany), then lyophilized for 2 days and reweighed. The mass loss was used to estimate solvent volume, and the dry residue was used to estimate solute mass. For salt-containing eluents, dried ${Na}_{2}{SO}_{4}$ must be subtracted either using a dried eluent blank at the same salt concentration or by calculating the expected salt mass from the measured residual solution volume. Without this correction, the dry residue would overestimate polymer mass. With salt correction, the method is defensible for correcting filter retention and water carryover. The calculated polymer concentration for each sample, obtained via this method, was entered into the SEC software, OmniSEC 5.12 (Malvern Panalytical, Worcestershire, UK) for subsequent quantitative analysis.

### 2.4. Quantitative Chromatographic Criteria

To compare eluent conditions objectively, standard chromatographic terminology was adopted to define quantitative criteria for chromatograms suitable for meaningful SEC-RI/RALS analysis. Peak symmetry and the signal-to-noise ratio were evaluated using common chromatographic system-suitability concepts, and the signal-to-noise criterion was interpreted consistently with “ICH Harmonised Guideline: Validation of Analytical Procedures Q2(R2)” [37]. These references were used to support the definitions and calculations of chromatographic-quality metrics. However, the present study was not intended to constitute pharmacopeial validation of the SEC method. The numerical limits used in this study were thus treated as study-specific operational acceptance limits for this screening experiment rather than as universal regulatory requirements.

The criteria were applied differently to the PEO standard and the alginate salt-screening experiments because the two analyses served different purposes. The PEO standard was used to assess system suitability and to verify molecular weight and polydispersity measurements. Hence, strict single-peak criteria, including peak symmetry, peak width, RI/RALS correspondence, baseline stability, signal-to-noise ratio, and repeatability, were appropriate. In contrast, alginate was evaluated as a polydisperse natural polysaccharide during mobile-phase salt screening. For this reason, alginate chromatograms were judged using peak-level chromatographic quality rather than by forcing every trace to satisfy narrow-standard peak-shape criteria. Thus, multimodal or shouldered alginate traces were not rejected solely because they were non-Gaussian. Instead, they were evaluated for baseline recovery, adequate signal-to-noise ratio, RI/RALS correspondence over the main polymer elution peak, and tailing. Gaussian fitting was not used as an acceptance criterion in this study because several traces were multimodal or convoluted and were therefore not adequately represented by a single Gaussian function. Importantly, SEC analysis does not require a Gaussian peak shape, especially for polydisperse polymers such as alginate, for which the elution profile may reflect the underlying molecular-weight distribution. Table 2 summarizes and compares the chromatographic criteria applied to each polymer, and Appendix B presents a detailed analysis of the quantitative assessments.

### 2.5. Data Analyses

Molecular-weight distributions, including the number-average molecular weight ($M_{n}$) and weight-average molecular weight ($M_{w}$) of samples, were determined using an integrated RI and RALS detectors configuration [17,38,39,40,41,42]. Data were processed utilizing OmniSEC 5.12 software (Malvern Panalytical). Because the coupled RI/RALS methodology calculates slice-by-slice molecular weight directly via the refractive index increment (dn/dc) rather than relying on conventional relative calibration, a single neutral PEO standard ($M_{w}=46\;kg/mol$) was sufficient to establish system detector relationships [16,17]. Consequently, the SEC system was standardized using the PEO standard prepared in 0.1 M ${Na}_{2}{SO}_{4}$.

For RI detection, the local polymer concentration in each chromatographic slice ($c_{i}$) is derived from the RI signal through the refractive index increment (n) per concentration (dn/dc), according to

(2) $$c_{i}\propto \Delta n_{i}/(dn/dc)$$

where $\Delta n_{i}$ represents the differential refractive index of slice i, which is a portion with molecules with molecular weight $M_{i}$ in the sample, and dn/dc is the specific refractive-index increment. For each sample, the polymer concentration value entered into software was the experimentally determined post-filtration concentration during sample preparation. This concentration, together with the programmed injection volume (100 µL), defined the injected polymer mass used for the analysis. The total injected mass is also subsequently determined by integrating this concentration profile across the elution peak, yielding a value consistent with the direct measurements described earlier. dn/dc  is calculated from the experimental RI response and the known injected sample mass. Specifically, the software integrates the baseline-corrected RI signal over the selected polymer peak region and relates the RI peak area to the amount of polymer injected. Because the RI response is proportional to polymer concentration and dn/dc, the known injected mass allows the software to solve for the sample-specific dn/dc value. The calculated dn/dc is then used to convert the RI signal into a concentration profile across the chromatographic peak. This concentration profile was combined with the RALS response to calculate slice-by-slice molecular weight ($M_{i}$) based on

(3) $$light-scattering\;intensity\propto c_{i}\times M_{i}\times {\left(dn/dc\right)}^{2}$$

The precise molecular weight of each individual chromatographic slice can be quantified, thereby generating a comprehensive molecular weight profile across the entire elution peak. Once the molecular weight of each slice is determined, $M_{n}$, $M_{w}$, and the polydipersity index (PDI) are obtained by

(4) $$M_{n}\equiv \frac{\Sigma N_{i}M_{i}}{\Sigma N_{i}}$$

(5) $$M_{w}\equiv \frac{\Sigma N_{i}M_{i}^{2}}{\Sigma N_{i}M_{i}}$$

(6) $$PDI\equiv \frac{M_{w}}{M_{n}}$$

where $N_{i}$ is the number of molecules in slice i.

A single PEO standard remains highly effective in standardizing the system even though its nominal molecular weight does not span the entire mass range of the target sample (e.g., alginate). The reference standard functions solely to establish essential inter-detector relationships (i.e., standardizing the SEC), whereas the molecular weight of a target sample (e.g., alginate) is calculated directly from its intrinsic scattering and concentration signals. Conversely, if conventional relative RI calibration were employed, a single PEO standard would fail to adequately encompass the higher molecular-weight distribution of the alginate.

Because the same polymer samples were analyzed under different eluent conditions, any salt-dependent variation in calculated $M_{n}$ or $M_{w}$ reflects changes in chromatographic behavior and detector-derived analysis rather than true changes in polymer molecular weight. Thus, the molecular-weight values reported here are interpreted as “apparent” SEC/RALS-derived molecular weights. For simplicity, $M_{n}$, $M_{w}$, and molecular weight are used throughout this manuscript to denote these apparent values unless explicitly stated otherwise. Since dn/dc depends on the polymer–solvent–temperature combination, such condition-specific dn/dc values are required for exact molecular weight determination for each condition.

### 2.6. Statistical Analysis

For the PEO reference standard, three to four replicate analyses were performed at each ${Na}_{2}{SO}_{4}$ concentration. $M_{n}$ and $M_{w}$ were analyzed separately and reported as the mean ± standard deviation. A one-way analysis of variance (ANOVA) was used to test the null hypothesis that the average molecular weights were independent of the ${Na}_{2}{SO}_{4}$ concentration, with statistical significance defined as p<0.05. When ANOVA indicated a significant salt-concentration effect, Tukey’s Honestly Significant Difference (HSD) post hoc test was applied to identify specific pairwise differences between salt concentrations. Because ANOVA and Tukey’s HSD test for group differences but do not directly establish monotonic concentration trends, additional trend analyses were performed using least-squares linear regression and Spearman rank correlation. These trend analyses were conducted both with all PEO eluent conditions included and after excluding the 0 M ${Na}_{2}{SO}_{4}$ condition to determine whether any apparent decreasing trend persisted among salt-containing eluents alone. Replicate injections were performed for PEO46k because this polymer serves as the standard for instrument calibration; consequently, its resulting molar mass and polydispersity index (PDI) values are method-critical. Because this specific material functions as a primary calibrant, maximizing chromatogram reproducibility and accuracy was important.

In contrast, the alginate experiments were designed as a high-resolution ${Na}_{2}{SO}_{4}$ concentration screen (prioritizing a wide range of concentrations over multiple replicates at fewer concentrations) to identify optimal eluent conditions rather than establish lot-specific molecular weights for the commercial alginate. Once the target eluent system is established, replicate alginate analyses are recommended for batch release, specification setting, or absolute molecular weight reporting, and most alginate salt conditions were analyzed without replicate measurements. Therefore, inferential statistical tests such as ANOVA and Tukey’s HSD were not applied to the alginate molecular-weight data. Instead, alginate results were evaluated descriptively by comparing chromatographic features, including peak shape; peak broadness; RI/RALS co-elution; tailing; late-eluting small-molecule or salt-related features; and the corresponding $M_{n}$, $M_{w}$, and polydispersity values. Consequently, alginate results are interpreted as descriptive evidence of salt-dependent chromatographic behavior rather than replicate-based statistical proof of differences between individual salt concentrations.

### 2.7. AI-Assisted Preparation of Schematic Figure

The spherical bead image used in Figure 1 was generated using ChatGPT 2.52 (OpenAI, San Francisco, CA, USA) from author-provided prompts requesting a grayscale, generic porous chromatographic bead for use in a conceptual schematic. The AI-generated image was used only as a visual representation of a generic porous size-exclusion chromatography packing particle. The remaining figure elements, including the magnified pore-network schematics, polymer-chain cartoons, annotations, labels, and panel arrangement, were prepared and edited by the authors. The AI-generated bead image was not derived from experimental microscopy, was not used for quantitative image analysis, and was not used to generate, process, or interpret chromatographic data. The final figure was reviewed and edited by the authors to ensure consistency with the experimental observations and mechanistic interpretation described in this report.

## 3. Results and Discussion

### 3.1. PEO Chromatograms and Molecular Weights

**Figure 2:**
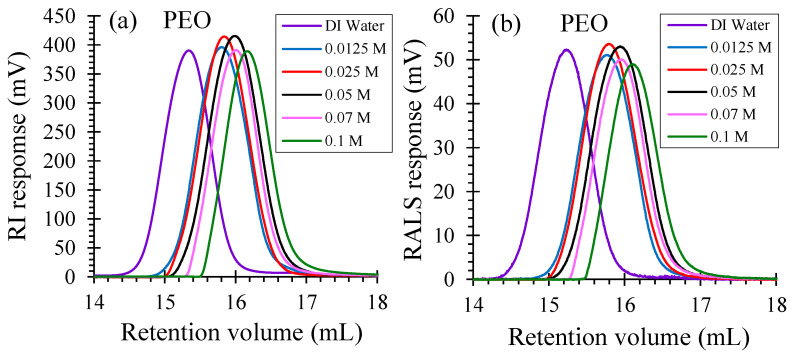
SEC chromatograms of PEO reference standard acquired in DI water and saline. (**a**) RI and (**b**) RALS traces are shown as a function of elution volume. PEO retained a single bell-shaped peak at all salt concentrations, indicating relatively uniform transport through the column. However, the peak position shifted with salt concentration, demonstrating that even a nominally neutral PEO reference is affected by aqueous mobile-phase composition. This salt-dependent shift was used to evaluate the suitability of the PEO chromatogram for system standardization and subsequent molecular-weight analysis.

Given its nominally neutral character, PEO was theoretically anticipated to function as an ideal standard with an elution profile independent of the mobile-phase ionic strength. However, as illustrated in Figure 2, the PEO RI and RALS peaks exhibit a progressive shift toward higher elution volumes as the salt concentration increases, all while maintaining a symmetric morphology. Based on quantitative analyses (Appendix B), all chromatograms exhibit minimal baseline drift (<0.05), low baseline noise (≪0.05), a robust signal-to-noise ratio (≫10), and a low normalized apex offset (<0.1, indicating RI and RALS coelution), with no observable shoulders. Although slight tailing is present (1.2 <SF<1.6), it remains within an acceptable symmetric range [43].

Although the variance between deionized water and the 0.1 M condition appears subtle, this measurable rightward shift indicates that PEO is not entirely interaction-free in an aqueous environment [44,45]. Crucially, a shift to higher elution volumes implies either greater effective pore penetration or a reduction in hydrodynamic volume at higher ionic strengths; conversely, had the salt induced stronger net exclusion, the peaks would have shifted to the left. A plausible explanation is that at very low ionic strengths, PEO maintains residual electrostatic or ion-dipole interactions, consistent with reports that PEO can complex with electrolytes to manifest a measurable cationic character [44,45]. In deionized water, these subtle interactions likely expand the hydrated coil or increase the apparent excluded volume. The addition of salt effectively screens these weak charge effects and modulates the hydration shell, thereby reducing the effective hydrodynamic volume and allowing the polymer chains to sample a greater portion of the pore volume. Because these interactions are mild and uniform across the macromolecular population, the peak remains symmetric, resulting in relatively consistent molecular-weight determinations despite the shift in retention.

Despite these shifts, the preservation of a symmetric, bell-shaped profile across the entire salt concentration range suggests that these nonideal effects are minimal. The absence of significant peak broadening or tailing indicates that, while the retention volume is slightly sensitive to ionic strength, the fundamental size-exclusion mechanism remains dominant and robust for the PEO standard.

**Figure 3:**
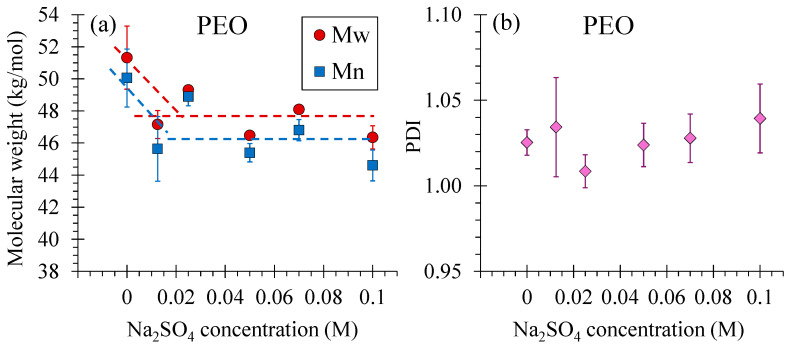
SEC-derived (**a**) $M_{n}$ and $M_{w}$, and (**b**) PDI values for PEO as a function of ${Na}_{2}{SO}_{4}$ concentration. Data are shown as mean ± standard deviation from three to four replicate analyses per salt concentration. The 0.00 M condition showed higher apparent molecular-weight values than several salt-containing eluents. One-way ANOVA and Tukey’s HSD analysis confirmed a statistically significant effect of salt concentration on both $M_{n}$ and $M_{w}$. However, trend analysis showed that the response was non-monotonic among the salt-containing eluents, indicating salt-dependent changes in apparent SEC/RALS-derived molecular weight rather than a simple continuous decrease with increasing ${Na}_{2}{SO}_{4}$ concentration. The dashed lines are intended solely as a visual guide for the eye and do not represent model predictions. All plotted PEO points include SD error bars. Where a bar is not visually obvious, it is smaller than or comparable to the plotted symbol.

Following system standardization with the PEO reference at 0.1 M ${Na}_{2}{SO}_{4}$, three to four replicate PEO analyses were performed at each salt concentration, and the resulting dn/dc, $M_{n}$, $M_{w}$, and PDI values are shown in Table A2 and Figure 3. Qualitatively, the rightward shift illustrated in Figure 2 of the chromatograms corresponds to a smaller apparent hydrodynamic size and therefore to lower apparent elution-based molecular weights. However, the relationship is not perfectly monotonic because RI–RALS molecular weight also depends on slice-by-slice concentration, light scattering, *dn*/*dc* handling, and integration boundaries. The range of experimentally determined dn/dc (0.136–0.159 mL/g) is generally wider and higher than literature values for various eluents (0.128–0.136 mL/g) [46,47,48,49], implying that concentration has a significant effect on dn/dc.

One-way ANOVA followed by Tukey’s HSD analysis showed that the salt concentration had a statistically significant effect on both average molecular weights, $M_{n}$ and $M_{w}$. Because ANOVA and Tukey’s HSD identify group differences but do not directly test monotonic concentration trends, additional trend analyses were performed. Visual inspection of the data in Figure 3 shows an initial decrease in the average molecular weights after the 0 M condition. However, after excluding the 0.00 M group, neither linear regression nor Spearman’s rank correlation showed a statistically significant decreasing trend among the salt-containing eluents. Linear regression gave p=0.1766 for $M_{n}$ and p=0.1576 for $M_{w}$, while Spearman’s rank correlation gave p=0.309 for $M_{n}$ and p=0.483 for $M_{w}$. These results indicate that, above 0 M Na_2_SO_4_, the molecular weights vary non-monotonically rather than decreasing systematically with increasing salt concentration. Hence, the data support a salt-dependent change in molecular weights, but they do not support a simple monotonic decrease of $M_{n}$ or $M_{w}$ across the saline eluents. Because each group contained only 3–4 replicates, these results should be interpreted as evidence of statistically detectable salt dependence under the tested conditions, while recognizing the limited replicate number and the non-monotonic concentration response.

The selection of the PEO chromatogram at 0.1 M ${Na}_{2}{SO}_{4}$ as the primary system standardization condition was not performed not simply because the calculated $M_{w}$ matched the nominal value of 46 kg/mol, which is expected because that chromatogram was used for standardization, but because this condition provided the most strongly screened and least distorted PEO peak among the tested conditions. The 0.1 M condition represents the most effectively screened state of the PEO reference, exhibiting the lowest sensitivity to mobile-phase variations among the concentrations tested. This condition yields a highly symmetric, unimodal peak with the maximal elution volume and minimal tailing, effectively mitigating the nonideal, low-ionic-strength effects observed in deionized water.

### 3.2. Alginate Chromatograms and Molecular Weights

**Figure 4:**
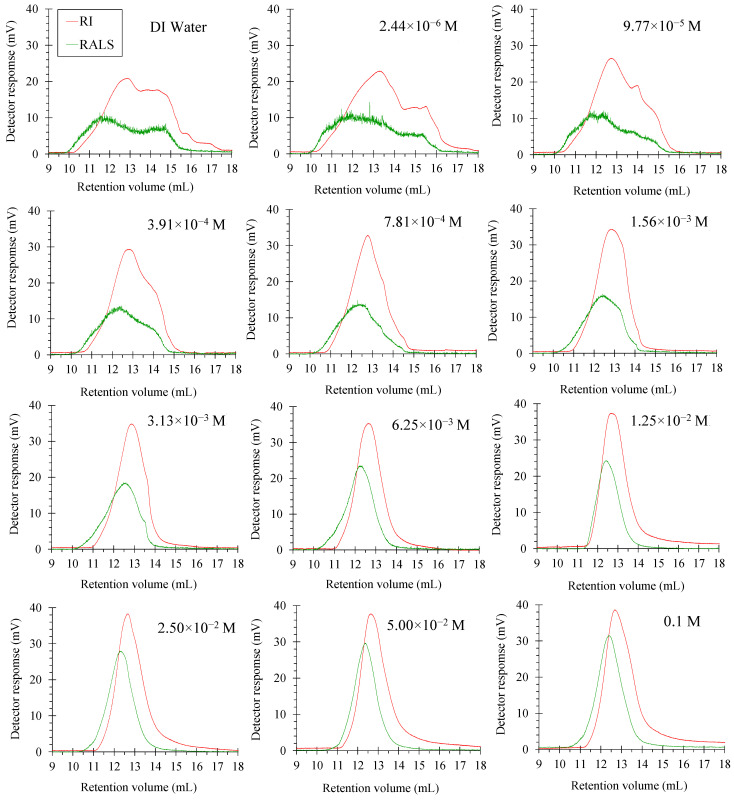
SEC chromatograms of alginate obtained in DI water and saline. RI traces are shown in red, and RALS traces are shown in green. In DI water and at a very low salt concentration, alginate produced broad, heterogeneous, and partially convoluted chromatograms, consistent with electrostatic chain expansion, heterogeneous pore access, and polymer–column interactions. Increasing ${Na}_{2}{SO}_{4}$ concentration progressively narrowed the main alginate peak and improved RI/RALS co-elution. The first condition that produced a clearly defined, approximately symmetric main peak was $6.25\times 10^{-3}\;M$ ${Na}_{2}{SO}_{4}$. At higher salt concentrations, right-side tailing and late-eluting features became more apparent, indicating that excessive salt did not further improve chromatographic fidelity.

Figure 4 illustrates alginate chromatograms at various salt concentrations. These are much more sensitive to salt concentration than the PEO reference chromatograms in Figure 2. In DI water and at very low salt concentration, neither the RI nor the RALS signal exhibits a clean, single, symmetric polymer peak. Instead, the main elution region is broad, heterogeneous, and partially convoluted, with shoulders and poorly resolved components. This behavior is characteristic of nonideal SEC of polyelectrolytes, in which elution is influenced not only by hydrodynamic size but also by electrostatic chain expansion, pore exclusion, adsorption/desorption, and stationary phase interactions [19,20]. For alginate specifically, ionic strength is known to affect chain flexibility and hydrodynamic behavior in SEC [26]. The noisy low-salt RALS traces are also consistent with poor chromatographic focusing and heterogeneous transport. In a low-ionic-strength eluent, alginate chains are electrostatically expanded because repulsion among carboxylate groups increases the effective hydrodynamic volume. As a result, some short and medium chains that would normally enter pores are partially excluded and elute earlier than expected, while another fraction interacts with pore entrances, bead surfaces, frits, or other wetted surfaces and elutes later. The coexistence of early elution by electrostatic exclusion and delayed elution by adsorption/desorption broadens the chromatogram and produces the convoluted peak shapes observed at low salt [19,20]. Thus, the low-salt molecular-weight values should be interpreted cautiously as apparent SEC/RALS-derived values rather than ideal size-exclusion molecular weights.

As the salt concentration increases, the main alginate peak becomes markedly narrower and more bell-shaped, indicating that much of the low-salt broadening was nonideal rather than simply a reflection of intrinsic dispersity. Added salt screens intramolecular electrostatic repulsion and reduces electrostatic polymer–surface interactions, allowing the chains to approach a screened-coil conformation and to access the pore volume more reproducibly [26,50]. Among the tested conditions, $6.25\times 10^{-3}\;M$ ${Na}_{2}{SO}_{4}$ is the first concentration at which the main RI and RALS peaks become sufficiently coherent and approximately symmetric, and it is therefore identified as the practical minimum effective salt concentration for reliable alginate SEC under the present conditions. To avoid over-interpreting this single-run alginate screen, the term minimum-effective salt concentration is used operationally rather than as a statistically validated optimum.

Peak symmetry and RI/RALS peak correspondence were used as system-suitability criteria for the narrow PEO standards because these standards were used to verify detector alignment, chromatographic band broadening, and molecular-weight/polydispersity calculations. In contrast, multimodal or shouldered traces were not rejected solely on that basis for alginate. Alginate is a polydisperse natural polysaccharide, and its SEC profile may reflect a broad or multimodal molecular-weight distribution. Thus, neither perfect peak symmetry nor strict unimodality was required for the alginate salt-screening experiment.

Instead, the alginate chromatograms were evaluated using operational peak-quality criteria, including formation of a dominant polymer elution peak detected by both RI and RALS, acceptable signal-to-noise and baseline recovery around the polymer peak, reduced salt-dependent convolution or shoulders, and absence of dominant late-eluting RI features or detector-inconsistent scattering features. Because RI and RALS respond differently to polydisperse samples, exact apex overlap was not required for alginate. Rather, RI/RALS correspondence was assessed by whether both detectors described the same main polymer elution peak after detector-delay correction (Appendix B). Single-peak metrics such as symmetry factor and fitted peak width were applied only to traces that could reasonably be described by one dominant peak, and they were not forced onto low-salt alginate traces that were clearly multimodal or convoluted. Signal-to-noise ratio and baseline stability, however, were evaluated over the full polymer peak (Appendix B), because these metrics do not require a Gaussian peak.

Using these criteria, the preferred condition was defined as the minimum effective ${Na}_{2}{SO}_{4}$ concentration, rather than the highest salt concentration tested or the condition giving the most Gaussian peak. Specifically, the selected condition was the lowest ${Na}_{2}{SO}_{4}$ concentration at which the alginate chromatogram changed from low-salt, salt-dependent convoluted behavior to an acceptable RI/RALS polymer peak. Under this evaluation, $6.25\times 10^{-3}\;M$ ${Na}_{2}{SO}_{4}$ was retained as the first acceptable condition. At this concentration, the RI/RALS polymer peak was substantially improved, with a decent signal-to-noise ratio (>100) and negligible baseline drift (<0.05) and baseline noise (<<0.05), acceptable baseline recovery around the main polymer peak, SF~1.5, and no dominant shoulder or late-eluting feature in the main polymer region. Increasing the ${Na}_{2}{SO}_{4}$ concentration above $6.25\times 10^{-3}\;M$ did not provide a clear additional improvement in the main polymer peak. Although the main peak remained relatively narrow at higher salt concentrations, right-side tailing and late-eluting features became more evident. For example, the symmetry factor increases significantly (SF>2), indicating the onset of peak tailing at $1.25\times 10^{-2}\;M$. We evaluated SF as an indicator of tailing, although optimizing peak symmetry was not the primary objective of this study.

This behavior suggests that once the dominant electrostatic polyelectrolyte effect has been suppressed, further salt addition does not necessarily improve SEC separation. Instead, higher-salt conditions may accentuate residual non-SEC effects, including weak adsorption/desorption, hydrogen bonding or polar interactions with the methacrylate-based packing, altered polymer hydration, and possible salt-related RI or injection artifacts [16,20]. Thus, $6.25\times 10^{-3}\;M$ ${Na}_{2}{SO}_{4}$ was selected as the preferred minimum-effective alginate eluent condition.

**Figure 5:**
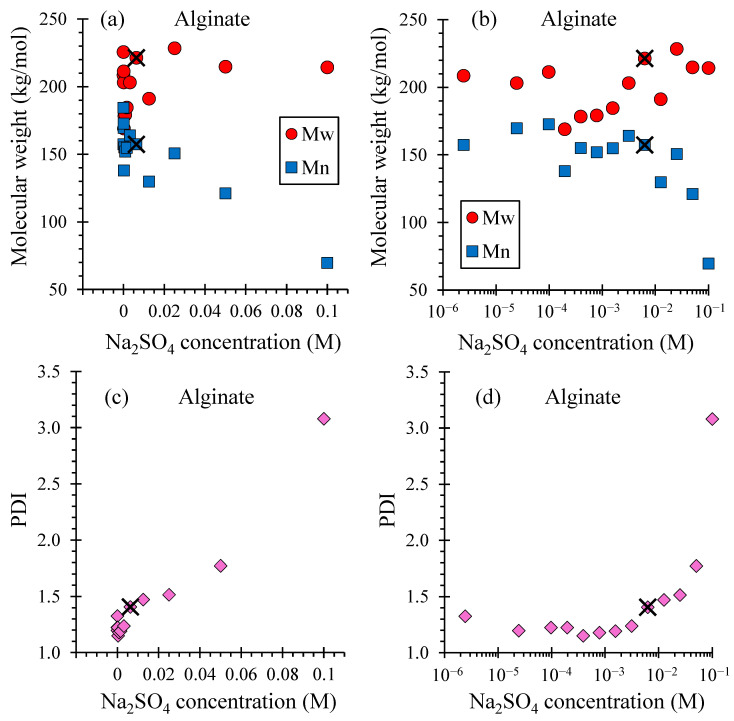
SEC-derived average molecular weights and polydispersity (PDI) for alginate as a function of ${Na}_{2}{SO}_{4}$ concentration. (**a**) Linear plot for $M_{n}$ and $M_{w}$, (**b**) semi-log plot for $M_{n}$ and $M_{w}$ excluding data at 0.00 M, (**c**) linear plot for PDI, and (**d**) semi-log plot for PDI. The “X” marker denotes $6.25\times 10^{-3}\;M$ Na_2_SO_4_, identified as the minimum-effective salt concentration based on chromatographic peak quality. $M_{w}$ remained comparatively stable across much of the salt range, whereas $M_{n}$ decreased more strongly at higher salt concentration. This behavior is consistent with increasing contributions from late-eluting low-molecular-weight fractions, tailing, or salt-related features, which affect $M_{n}$ more strongly than $M_{w}$, resulting in increasing PDI with salt concentration. No error bars are shown for alginate because the alginate series was a single-run high-resolution screen. The values guide protocol selection rather than define an exact alginate molar mass.

Following system standardization with the PEO reference at 0.1 M ${Na}_{2}{SO}_{4}$, molecular weights were quantified for alginate across various salt concentrations. Defining integration boundaries proved challenging at low ionic strengths due to the presence of poorly resolved or multimodal peak structures. Consequently, the integration range was established based on the RALS signal, spanning from the point of initial deviation from the baseline to the point of return to the baseline. These limits were then systematically applied to the corresponding RI signals. The resulting dn/dc, molecular-weight data, and elution trends are presented in Table A3 and Figure 5, in which the condition marked with “X” at $6.25\times 10^{-3}\;M$ ${Na}_{2}{SO}_{4}$ represents the first salt concentration at which the main RI and RALS features appeared sufficiently coherent and SEC-like.

The range of experimentally determined dn/dc (0.129–0.182 mL/g) is generally wider and higher than literature values for various eluents (0.150–0.165 mL/g, [8,51,52,53,54]). The resulting $M_{n}$ decreased more strongly with salt concentration than $M_{w}$. This difference is physically meaningful. In SEC/light-scattering analysis, $M_{n}$ is highly sensitive to low-molecular-weight slices because it is number-weighted, while $M_{w}$ is more strongly influenced by the mass contribution of higher-molecular-weight species. In slice-based terms, $M_{n}$ contains a strong contribution from terms proportional to $c_{i}/M_{i}$, so even a modest late-eluting low-molecular-weight tail can substantially lower $M_{n}$. By contrast, $M_{w}$ is weighted toward larger chains and is less affected by low-molecular-weight material unless that fraction contributes substantial mass [17]. This interpretation agrees with the chromatograms (Figure 4). At low salt concentrations, small-chain and interaction-derived features appear embedded within a broad, convoluted main peak. As the salt concentration increases, the main peak narrows, but late-eluting shoulders or tails become increasingly visible. Above $6.25\times 10^{-3}\;M$ ${Na}_{2}{SO}_{4}$, especially at 0.05–0.1 M, the chromatograms show more pronounced right-side tailing and late-eluting features. This situation explains the strong increase in PDI, which is a mathematical consequence of a decreasing $M_{n}$ combined with a relatively stable $M_{w}$.

Even if there are multimodal or convoluted peaks, we can still obtain molecular weights. We can still obtain an overall apparent molecular weight distribution and integrated average values, but we cannot necessarily assign a separate, accurate molecular weight to each unresolved component unless the components are chromatographically resolved or deconvoluted with additional assumptions. If two polymer populations coelute in the same chromatographic slice, RALS gives a scattering-weighted average for that slice. The overall $M_{w}$ can still be meaningful, but $M_{n}$ and polydispersity are more sensitive to separation quality, and poor separation can overestimate $M_{n}$ and underestimate polydispersity [17].

Table A3 is provided to document the salt-screening trend. These data should not be used as replicate-based statistical proof of alginate molar mass. The recommended protocol is to repeat the use of alginate at the selected eluent when exact molar mass or batch-to-batch comparisons are required.

### 3.3. Comparison of Alginate and PEO Behavior

PEO and alginate show markedly different chromatographic behavior in Figure 2 and Figure 4 because PEO is flexible and only weakly interactive in water, whereas alginate is a strongly anionic polysaccharide with mannuronic/guluronic compositional heterogeneity, strong counterion sensitivity, and a greater tendency toward non-SEC interactions than PEO. PEO thus maintains a bell-shaped peak across the entire salt-concentration range, as shown in Figure 2, whereas alginate is severely distorted in DI water and at very low salt concentration, as shown in Figure 4.

In Figure 4, the main alginate peak position appears less salt-sensitive than the PEO peak position illustrated in Figure 2, whereas the alginate peak shape changes much more strongly. For alginate, several competing effects, such as improved pore access, partial chain contraction, deconvolution of overlapped components, and changing contribution of low-molecular-weight species, may offset each other, so the apex does not move as dramatically as the PEO apex. For PEO, the peak shape is already good, so the visible salt effect is mainly the shift in elution volume.

In Figure 4, the apparent inconsistency between narrower alginate peaks and nearly unchanged PDI up to $6.25\times 10^{-3}\;M$ ${Na}_{2}{SO}_{4}$ is not contradictory. Visual peak narrowing does not automatically force PDI to decrease because PDI is determined by chromatographic moments and the full integration window, not by peak width alone. If $M_{n}$ and $M_{w}$ shift together or low-level tails remain in the integration window, PDI may stay similar, as seen in Figure 3 for PEO, while the central chromatogram becomes visibly narrower. At higher salt concentrations, tailing and the late peak disproportionately depress $M_{n}$ relative to $M_{w}$, so PDI increases more strongly, as reflected in Figure 5.

### 3.4. Practical Selection of Standardization and Working Eluent Concentrations

Two decisions must be separated: the PEO standardization condition used to calibrate detector relationships and the working salt concentration used to analyze the target polymer. For the first decision, 0.1 M ${Na}_{2}{SO}_{4}$ is preferred for PEO because it gives the cleanest, most screened PEO behavior and minimizes low-ionic-strength effects. For the second decision, $6.25\times 10^{-3}\;M$ ${Na}_{2}{SO}_{4}$ is preferred for alginate because it is the minimum concentration that yields a credible main peak while avoiding the larger late salt peak and stronger tailing seen at 0.0125–0.1 M. Thus, the same salt concentration need not be optimal for standardization and sample analysis.

It is acceptable to standardize with PEO reference of $M_{w}=46\;kg/mol$ even though alginate molecular weights are higher, because RI–RALS analysis uses the standard to establish detector constants, alignment, and band broadening, while sample molecular weight comes from the sample’s own scattering and concentration signals. Exact overlap between standard and analyte molecular-weight ranges is not mandatory. If the workflow were based only on conventional elution-volume calibration, a multi-standard calibration spanning the alginate range would be required.

### 3.5. Extending the Workflow to Other Polymers and Column Materials

The lesson is broader than alginate. Users analyzing other water-soluble polymers should identify the stationary-phase chemistry and then screen ionic strength accordingly. Common aqueous SEC column materials include methacrylate/polymethacrylate or PMMA-type packings, polyhydroxymethacrylate packings, poly(vinyl alcohol)-modified packings, silica-based hydrophilic or diol-bonded packings, and soft gel media such as agarose or dextran. Surface polarity, hydrogen-bonding character, residual charge, and pore architecture all influence non-SEC interactions. A salt concentration that works on one column chemistry may not be optimal on another.

The recommended workflow is (i) choose a minimally interacting (i.e., neutral) standard and test it across a small salt series; (ii) choose the standardization condition that gives the cleanest and most internally consistent standard peak with a few repeats; (iii) run the target water-soluble polymer across a broader salt series; (iv) identify the minimum effective salt concentration that yields a single symmetric main RI/RALS peak and acceptable baseline; (v) verify that late peaks are not injection- or salt-artifact peaks by running blanks and rinse controls; (vi) repeat SEC runs at the minimum effective salt concentration; and (vi) re-optimize (i.e., repeating (iv) and (v)] if polymer structure changes substantially after chemical modifications such as methacrylation, dopamine grafting, oxidation, sulfation, or deacetylation. This workflow can be used for a broader range of water-soluble polymers, including alginate derivatives (Alg-MA), gelatine derivatives (e.g., Gel-MA), HAMA, chitosan derivatives, pectin, carrageenan, and other charged or highly hydrated biopolymers. For routine quality control or for reporting the absolute molar mass of a particular polymer lot, the final selected eluent should then be confirmed by replicate injections, salt-matched blanks, and polymer-specific *dn/dc* measurement.

## 4. Conclusions

Aqueous SEC (GPC) of water-soluble polymers is strongly affected by eluent ionic strength when polymer–column interactions and polyelectrolyte chain expansion are present. The neutral PEO reference maintained a single bell-shaped RI/RALS peak across the ${Na}_{2}{SO}_{4}$ range, but its elution volume and SEC/RALS-derived molecular weights still varied with salt concentration, indicating that even a nominally neutral reference polymer is sensitive to aqueous mobile-phase conditions.

Alginate (anionic polyelectrolyte) showed a much stronger salt dependence. In DI water and very low salt, the chromatograms were broad, noisy, and convoluted, consistent with electrostatic chain expansion, heterogeneous pore access, and adsorption/desorption at bead or pore surfaces. Adding ${Na}_{2}{SO}_{4}$ screened these interactions and progressively narrowed the main alginate peak. The first condition that produced a clear, approximately symmetric RI/RALS main peak was $6.25\times 10^{-3}\;M$ ${Na}_{2}{SO}_{4}$, which is thus identified as the preferred minimum-effective salt concentration for this alginate/column/instrument system. Higher salt concentrations were not automatically better. Above $6.25\times 10^{-3}\;M$ ${Na}_{2}{SO}_{4}$, the main peak showed increasing tailing and late-eluting features, likely from residual non-electrostatic interactions, deeper pore access by low-molecular-weight fractions, RI/salt artifacts, or delayed desorption. These late contributions disproportionately lowered $M_{n}$ while $M_{w}$ remained comparatively stable, leading to an inflated polydispersity. Overall, the results support a minimum-effective-salt strategy for aqueous SEC: use enough salt to restore reproducible size-based separation, but avoid unnecessarily high salt concentration, which can introduce tailing, baseline artifacts, or operational concerns.

## Figures and Tables

**Table 1:** Common salts/additives used in aqueous SEC with typical functions, practical advantages, limitations, and hardware implications.

Additive	Typical Role in Aqueous SEC	Advantages	Disadvantages/Hardware Cautions
Sodium sulfate (Na_2_SO_4_)	Ionic-strength modifier for water-soluble polymers, and useful when chloride is undesired. Used in this study for PEO and alginate screening.	Efficient electrostatic screening, chloride-free, and used in aqueous alginate SEC studies [25,26,28].	Nonvolatile; dried deposits can accumulate at the injector/fill port and create late RI salt peaks; high concentrations can increase baseline/refraction artifacts and backpressure. Sulfate is generally less aggressive than chloride, but crystallization/fouling still requires flushing.
Sodium nitrate (NaNO_3_)	Neutral electrolyte often used for aqueous SEC of polyelectrolytes.	Chloride-free, widely used as a mobile-phase salt for polyelectrolyte SEC method development [20].	Nonvolatile and can still form deposits or late salt peaks. Nitrate is less notorious than chloride for pitting stainless steel, but oxidizing nitrate media can complicate compatibility for some samples and alloys.
Sodium chloride (NaCl)	Strong ionic screening, biologically familiar conditions, sometimes used when protein or biopolymer solubility requires physiological ionic strength.	Simple, inexpensive, and effective at suppressing charge effects; useful when pH and osmotic conditions matter.	Chloride-containing eluents are the most concerning for stainless-steel tubing, valves, frits, and injector parts because chloride accelerates pitting and passive-film breakdown [29,30,31], and overall corrosion.
Phosphate-buffered saline (PBS)	Provides pH control together with ionic strength.	Useful when polymer charge state depends strongly on pH and buffering is needed; common for proteins and polysaccharides [16].	Can precipitate with multivalent cations and nonvolatiles; leaves deposits if dried; may promote salt-out or secondary interactions in some SEC systems at higher ionic strength [16].
Ammonium acetate (NH_4_CH_3_CO_2_)	Volatile salt/buffer sometimes chosen for downstream mass spectrometry (MS) compatibility or easier removal.	More removable than inorganic sodium salts, useful when sample recovery or downstream compatibility matters.	At modest concentrations, it may not screen strong polyelectrolyte interactions sufficiently; buffering capacity is limited compared with inorganic salts.
Sodium azide (NaN_3_)	Low-concentration preservative added to inhibit microbial growth; not a primary screening salt.	Helpful for maintaining aqueous eluents and columns during storage.	Toxic; not a substitute for ionic-strength optimization. Waste handling and compatibility procedures are required.

**Table 2:** Recommended chromatographic criteria for the PEO standard and alginate salt screen.

Metric	Standard (e.g., PEO)	Sample (e.g., Alginate)	Remarks
Baseline stability	Yes	Yes	Applies to all chromatograms.
Baseline noise and S/N	Yes	Yes	For Sample, calculate over the full polymer peak.
RI/RALS correspondence	Yes	Yes	Standard should show close RI/RALS alignment after detector-delay correction. Sample only needs RI and RALS to describe the same polymer peak.
Shoulder/secondary feature ratio	Unnecessary	Yes	Important for Sample because the salt effect appears as shoulders, convolution, tailing, and late features.
Late-eluting artifact ratio	Unnecessary	Yes	Important for explaining why higher salt was not selected.
Peak symmetry factor	Yes	Only for one dominant peak	Appropriate for narrow standard. Not forced onto low-salt Sample traces that are convoluted or multimodal.
Gaussian fitting	Unnecessary	No	Optional for Standard. Avoid forcing a natural, polydisperse polymer into a single Gaussian model.
Molecular weights and PDI repeatability	Yes	Optional	Standard is repeated because molar mass/PDI verification is system-critical. Sample repeats are needed only for exact values.

## Data Availability

The original contributions presented in this study are included in the article. Further inquiries can be directed to the corresponding author.

## Appendix A. Operational Conditions for SEC

**Table A1:** Operational conditions used in the present study.

Parameter	Condition
Instrument	Malvern TDA305 with GPCmax pump/autosampler
Detectors	RI and RALS
Columns	Malvern Panalytical/Viscotek A3000 and A4000 aqueous GPC/SEC columns (300 × 8 mm each) connected in series. Exclusion limits: 100 kg/mol (A3000) and 1000 kg/mol (A4000) based on pullulan.
Stationary phase	A-series methacrylate/PMMA-type aqueous porous packing. A3000 provides lower-to-intermediate-size resolution, and A4000 extends the usable range toward larger water-soluble polymers
Column and detector temperature	35 °C
Flow rate	0.5 mL/min
Eluent	$\mathrm{DI}\;\mathrm{water}\;\mathrm{containing}\;{Na}_{2}{SO}_{4}$ from 0 to 0.1 M
Sample concentration	PEO: 5 mg/mL in the corresponding eluent alginate: 1 mg/mL in the corresponding eluent
Filtration	0.2 µm syringe filter before injection
Sample injection volume	100 µL
Software	Malvern OmniSEC 5.12
Replicates	PEO: 3 to 4 replicate runs at each salt concentration alginate: single runs across a broader salt series

## Appendix B. Quantitative Criteria

### Appendix B.1. Baseline Stability

First of all, the baseline of chromatograms should be stable. For each RI and RALS trace, two baseline windows are selected outside the polymer elution peak. The pre-peak baseline is taken before polymer elution, and the post-peak baseline is taken after the polymer peak. When a late salt or injection artifact is present, the post-peak window is chosen either before the artifact or after the artifact, depending on whether the purpose is to evaluate the main polymer peak or total baseline recovery. A straight-line baseline is fitted between the mean pre-peak and post-peak responses and subtracted from the chromatogram. Baseline stability (i.e., drift [55] and noise [56]) is then obtained as follows:

(A1) $$Baseline\;drift\;\equiv \frac{\left|{\bar{y}}_{post}-{\bar{y}}_{pre}\right|}{H_{polymer}}<0.05$$

(A2) $$Baseline\;noise\equiv \frac{\sigma_{baseline}}{H_{polymer}}<0.05$$

where “pre” means before the polymer peak, “post” means after the polymer peak; $\bar{y}$ is the average detector signal; $\sigma_{baseline}$ is the standard deviation of the baseline signal, which is obtained from the pre-peak and/or post-peak baseline region after subtracting the fitted baseline; and $H_{polymer}$ is the highest height of the polymer peak from the baseline. The 5% tolerance is not a universal pharmacopeial requirement; it is an operational acceptance criterion used here to compare SEC salt-screening conditions.

### Appendix B.2. Signal-to-Noise Ratio

The signal-to-noise ratio (S/N) is not a Gaussian peak metric and can be evaluated for both symmetric and asymmetric polymer peaks. The calculation method must be stated. When RMS baseline noise is used, S/N [57] was calculated as

(A3) $$\frac{S}{N}\equiv \frac{H_{peak}}{\sigma_{baseline}}$$

When peak-to-peak baseline noise is used, an alternative chromatographic convention is

(A4) $$\frac{S}{N}\equiv \frac{{2H}_{peak}}{h}$$

where h is the peak-to-peak baseline noise measured in an appropriate baseline window. For the present screening comparison, the operational quantitation criterion is as follows [37,58]:

(A5) $$\frac{S}{N}>10$$

For alginate, S/N is evaluated over the full polymer peak. It is not necessary for the peak to be Gaussian, symmetric, or strictly unimodal.

### Appendix B.3. RI/RALS Correspondence (Coelution)

RI and RALS signals may appear shifted because the detectors are physically arranged in series [59]. In this study, RALS preceded RI. Thus, detector delay was corrected using the narrow PEO standard before RI/RALS correspondence was evaluated. For a narrow standard, the RI and RALS apex positions should be closely aligned after detector-delay correction:

(A6) $$\Delta V_{apex}\equiv |V_{apex,RI}-V_{apex,RALS}|$$

where $V_{apex,RI}$ and $V_{apex,RALS}$ are the elution volumes at which the RI and RALS signals reach their maxima. For the PEO standard, the normalized apex offset was used as a strict system-suitability criterion:

(A7) $$\frac{\Delta V_{apex}}{W_{0.5}}\leq 0.1$$

For alginate [28], exact RI/RALS apex overlap was not required because RI primarily follows concentration, whereas RALS is more strongly weighted toward the high-molar-mass fraction. Instead, RI/RALS correspondence is judged by whether both detectors described the same main polymer elution peak after detector-delay correction, without a separate dominant RALS-only aggregate feature or RI-only late artifact.

### Appendix B.4. Dominant Polymer-Peak/Shoulder Criterion

Before applying single-peak metrics such as symmetry factor or fitted peak width, the chromatogram was evaluated to determine whether it could reasonably be described by one dominant peak or polymer peak [17]. The RI trace is used as the primary concentration-sensitive signal. RALS is used for confirmation, but an exact match between RI and RALS peak shapes is not required for alginate because light scattering is strongly weighted toward the higher-molar-mass fraction. The relative size of the shoulder can be obtained to judge how dominant the shoulder is, and a shoulder or secondary feature smaller than 15% of the main polymer response is treated as non-dominant for operational comparison:

(A8) $$\frac{H_{shoulder}}{H_{polymer}}<0.15\;or\;\frac{A_{shoulder}}{A_{polymer}}<0.15$$

where $A_{polymer}$ is the area of the polymer peak, and $H_{shoulder}$ and $A_{shoulder}$ are the height and area of any shoulder or secondary feature in the main polymer elution region, respectively. The 15% value is a predefined operational threshold for this screening comparison and should not be interpreted as a universal SEC requirement.

### Appendix B.5. Late-Peak/Salt-Artifact Criterion

Because high salt can create late RI artifacts or enhance tailing, late-eluting features were quantified separately from the main polymer peak [26]. The late-feature ratio was calculated using peak height or integrated area:

(A9) $$\frac{H_{late}}{H_{polymer}}<0.1\;or\;\frac{A_{late}}{A_{polymer}}<0.1$$

where the subscript “late” refers to features at high retention volume, especially features observed mainly in RI without a corresponding RALS signal. A late-feature ratio above the operational threshold was interpreted as evidence that increasing the salt concentration did not further improve the SEC separation and could introduce salt-related RI, injection, hydration, adsorption/desorption, or other non-SEC effects.

### Appendix B.6. Peak Width and Symmetry

Peak width is used as a comparative chromatographic-quality metric [60]. For alginate, which is a polydisperse natural polysaccharide, peak width was interpreted as the width of the polymer elution peak rather than the width of a forced Gaussian fit. When a fixed fractional-height criterion is used, the width is calculated as

(A10) $$W\equiv V_{right}-V_{left}$$

where $V_{right}$ and $V_{left}$ are the elution volumes where the polymer elution region ends and starts, respectively. For a narrow PEO standard, the full width at half height, $W_{0.5}$, or the width at 5% peak height, $W_{0.05}$, can be applied as a conventional single-peak metric:

(A11) $$W_{0.5}\equiv V_{right,0.5}-V_{left,0.5}$$

(A12) $$W_{0.05}\equiv V_{right,0.05}-V_{left,0.05}$$

where subscripts “0.5” and “0.05” mean the values measured at 50% and 5% of the peak height, respectively. The pharmacopeial-style peak symmetry factor [61] was defined as

(A13) $$SF\equiv \frac{W_{0.05}}{2f_{0.05}}\;\left\{\begin{array}{c} =1:a\;\mathrm{symetric}\;\mathrm{peak}\;\\ >1:a\;\mathrm{tailing}\;\mathrm{peak}\;\\ <1:a\;\mathrm{fronting}\;\mathrm{peak}\; \end{array}\right.\;$$

where $f_{0.05}$ is the distance from the leading-edge 5% height point to the perpendicular line through the peak maximum. Chromatography references commonly use $A_{s}$ for this quantity, but SF is used here to avoid confusion with the peak area, A.

For narrow, well-behaved standards such as PEO, PEG, pullulan, or polystyrene, peak symmetry is useful because it indicates appropriate column performance, injection conditions, mobile-phase composition, detector-delay correction, and baseline handling. A practical symmetry-factor range for system-suitability screening follows [43]:

(A14) 0.8<SF<1.8

For alginate, however, neither perfect peak symmetry nor strict unimodality was required. A broad, asymmetric, or shouldered alginate trace may reflect the true molecular-weight distribution of a natural polysaccharide as well as chromatographic band broadening. Nevertheless, severe tailing, fronting, salt-dependent shoulders, or late artifacts were interpreted as evidence of nonideal SEC behavior, such as electrostatic interaction with the column, aggregation or disaggregation, adsorption, or baseline/salt mismatch. Therefore, the symmetry factor was calculated for alginate only when the trace contained one dominant polymer peak. It was not forced onto low-salt traces that were clearly multimodal or convoluted. A salt condition was considered less preferred when the main alginate peak shows substantial right-side tailing, defined operationally as

(A15) SF>2

when SF was applicable, or when a late-eluting feature exceeded 0.1 of the main peak height or main peak area, these limits were used as study-specific screening criteria.

### Appendix B.7. Gaussian Fitting

Gaussian fitting was not used as an acceptance criterion in this study because several traces were multimodal or convoluted and were therefore not adequately represented by a single Gaussian function. Importantly, SEC analysis does not require a Gaussian peak shape, especially for polydisperse polymers such as alginate, for which the elution profile may reflect the underlying molecular-weight distribution.

## Appendix C. Experimental Data

**Table A2:** Mean $M_{n}$, $M_{w}$, PDI, and dn/dc values for PEO across various ${Na}_{2}{SO}_{4}$ concentrations, including the associated standard deviations. These replicate values support the PEO46k standardization and method-setting step.

Sodium Sulfate Concentration (M)	Ionic Strength (M)	$M_{n}$ (kg/mol)	$M_{w}$ (kg/mol)	PDI	dn/dc
0.00	0.00	50.1 ± 1.8	51.3 ± 2.0	1.03 ± 0.01	0.156 ± 0.0085
1.25 × 10^−2^	3.75 × 10^−2^	45.6 ± 2.0	47.2 ± 0.9	1.03 ± 0.03	0.139 ± 0.0167
2.50 × 10^−2^	7.50 × 10^−2^	48. 9 ± 0.6	49.3 ± 0.2	1.01 ± 0.01	0.153 ± 0.0037
5.00 × 10^−2^	1.50 × 10^−1^	45.4 ± 0.6	46.5 ± 0.3	1.02 ± 0.01	0.159 ± 0.0027
7.00 × 10^−2^	2.10 × 10^−1^	46.8 ± 0.7	48.1 ± 0.2	1.03 ± 0.01	0.136 ± 0.0004
1.00 × 10^−1^	3.00 × 10^−1^	42.0 ± 1.0	46.4 ± 0.7	1.10 ± 0.02	0.140 ± 0.0059

**Table A3:** $M_{n}$, $M_{w}$, PDI, dn/dc values for alginate across various ${Na}_{2}{SO}_{4}$ concentrations. Values are single-run screening results and are therefore reported without SD.

Sodium Sulfate Concentration (M)	Ionic Strength (M)	$M_{n}$ (kg/mol)	$M_{w}$ (kg/mol)	PDI	dn/dc
0.00	0.00	184.3	225.5	1.22	0.165
2.44 × 10^−6^	7.32 × 10^−6^	157.3	208.5	1.33	0.182
2.44 × 10^−5^	7.32 × 10^−5^	169.5	203.0	1.20	0.178
9.77 × 10^−5^	2.93 × 10^−4^	172.5	211.3	1.23	0.174
1.95 × 10^−4^	5.85 × 10^−4^	138.0	168.9	1.22	0.164
3.91 × 10^−4^	1.17 × 10^−3^	154.9	178.3	1.15	0.148
7.81 × 10^−4^	2.34 × 10^−3^	151.9	179.2	1.18	0.131
1.56 × 10^−3^	4.68 × 10^−3^	154.7	184.5	1.19	0.157
3.13 × 10^−3^	9.39 × 10^−3^	164.0	203.0	1.24	0.154
6.25 × 10^−3^	1.88 × 10^−2^	157.3	221.3	1.41	0.152
1.25 × 10^−2^	3.75 × 10^−2^	129.7	191.1	1.47	0.129
2.50 × 10^−2^	7.50 × 10^−2^	150.6	228.3	1.52	0.145
5.00 × 10^−2^	1.50 × 10^−1^	121.1	214.7	1.77	0.136
1.00 × 10^−1^	3.00 × 10^−1^	69.6	214.2	3.08	0.156
